# A Hierarchical Graph Learning Model for Brain Network Regression Analysis

**DOI:** 10.3389/fnins.2022.963082

**Published:** 2022-07-12

**Authors:** Haoteng Tang, Lei Guo, Xiyao Fu, Benjamin Qu, Olusola Ajilore, Yalin Wang, Paul M. Thompson, Heng Huang, Alex D. Leow, Liang Zhan

**Affiliations:** ^1^Department of Electrical and Computer Engineering, University of Pittsburgh, Pittsburgh, PA, United States; ^2^Mission San Jose High School, Fremont, CA, United States; ^3^Department of Psychiatry, University of Illinois Chicago, Chicago, IL, United States; ^4^Department of Computer Science and Engineering, Arizona State University, Tempe, AZ, United States; ^5^Imaging Genetics Center, University of Southern California, Los Angeles, CA, United States

**Keywords:** multimodal brain networks, human connectome project, graph learning, interpretable AI, adult self-report score

## Abstract

Brain networks have attracted increasing attention due to the potential to better characterize brain dynamics and abnormalities in neurological and psychiatric conditions. Recent years have witnessed enormous successes in deep learning. Many AI algorithms, especially graph learning methods, have been proposed to analyze brain networks. An important issue for existing graph learning methods is that those models are not typically easy to interpret. In this study, we proposed an interpretable graph learning model for brain network regression analysis. We applied this new framework on the subjects from Human Connectome Project (HCP) for predicting multiple Adult Self-Report (ASR) scores. We also use one of the ASR scores as the example to demonstrate how to identify sex differences in the regression process using our model. In comparison with other state-of-the-art methods, our results clearly demonstrate the superiority of our new model in effectiveness, fairness, and transparency.

## 1. Introduction

Understanding brain structural and functional changes and its relationship to other phenotypes (e.g., behavior and demographical variables or clinical outcomes) are of prime importance in the neuroscience field. One of the key research directions is to use neuroimaging data for predictive or regression analyses and identify phenotype-related imaging biomarkers. Many previous studies (Rusinek et al., 2003; Sabuncu et al., 2015; Seo et al., 2015; Duffy et al., 2018; Kim et al., 2019) focus on predicting phenotypes using imaging features from voxels or region-of-interests (ROIs). However, increasing evidences show that most of the phenotypes are the outcomes of the interactions among many brain regions (Lehrer, 2009; Van Den Heuvel et al., 2012; Sporns, 2013; Mattar and Bassett, 2019), therefore, using brain network for this prediction task attracts more and more attentions. Brain network (Sporns et al., 2004; Power et al., 2010; Sporns, 2011) represents a 3D brain graph model, comprising the nodes and the edges connecting to the nodes. The nodes are brain ROIs and the edges can be defined using diffusion-MRI derived fiber tracking or functional-MRI-derived correlation. Brain network has the potential to gain system-level insights into the brain dynamics related to those phenotypes.

Many studies have been conducted to relate brain networks to behavioral, clinical measures or demographical variables and identify the most predictive network features (Eichele et al., 2008; Uddin et al., 2013; Brown et al., 2017; Beaty et al., 2018; Tang et al., 2019, 2022; Li C. et al., 2020). However, most of these studies (Chennu et al., 2017; Li et al., 2017; Warren et al., 2017; Du et al., 2019; D́ıaz-Arteche and Rakesh, 2020; Kuo et al., 2020) focus on exploring correlations between the pre-defined network features (e.g., clustering coefficient, small-worldness, characteristic path length, etc.) and the measures to be predicted (such as cognitive impairment, biological variables, behavior profile, psychopathological scores, etc.). This may be sub-optimal since those derived brain network features contain less information than the original networks and may ignore important brain network attributes. Although using the entire brain network for the task can solve this issue, it will introduce another challenge in how to handle the high dimensional network data during the task. Obviously, the traditional linear regression method may not be a good choice and more advanced methods (Székely et al., 2007; Székely and Rizzo, 2009; Simpson et al., 2011, 2012; Varoquaux and Craddock, 2013; Craddock et al., 2015; Dai et al., 2017; Wang et al., 2017; Zhang et al., 2019b; Xia et al., 2020; Lehmann et al., 2021; Tomlinson et al., 2021) have been proposed for this purpose. Additionally, recently years have witnessed a great success in the deep learning tools which have been widely used to discover the biological characteristics of brain network-phenotype associations (Hu et al., 2016; Ju et al., 2017; Mirakhorli et al., 2020).

To analyze the complex network data (e.g., brain networks), deep graph learning techniques (Kipf and Welling, 2016; Hamilton et al., 2017; Veličković et al., 2017; Gao et al., 2018; Zhang and Huang, 2019; Zhang et al., 2019a) have gained significant attention. A typical category of deep graph learning techniques are the graph neural networks (GNNs), which are proposed based on the message passing mechanism. In general, GNNs can be summarized as (1) message aggregation across nodes and (2) message transformation (e.g., non-linear transformation) as updated node features. A graph convolution operation in GNNs enables each graph node to aggregate information from its neighbor nodes. Generally, one graph convolution layer can enable the graph node to aggregate local information from one-hop neighbors (i.e., directly connected nodes), while stacked graph convolution layers may enable the graph node to aggregate higher-level information from multi-hops neighbors (Dehmamy et al., 2019), where richer semantic information can be found. However, when stacking too many graph convolution layers, not only the effective information will be captured but also much noise will be introduced, which will break the network representation (Li et al., 2018; Chen et al., 2020). Therefore, an important issue for current graph learning methods is how to effectively capture the higher-level brain network features. Another issue for current graph learning techniques is that the models are not easy to interpret. Although many existing graph learning methods may well achieve good predictive performances for certain tasks (e.g., classification of diseases or prediction of clinical scores), they might be difficult to provide meaningful biological explanations or heuristic insights into the results (Wee et al., 2019; Xuan et al., 2019; Li Y. et al., 2020; Wang et al., 2021). This should be attributed to the black-box nature of the neural networks. Although it is easy to know what the neural network predicts (i.e., the output of the black-box model), it is difficult to understand how the neural networks make the decision (i.e., heuristic intermediate results inside the black box). To address these, a few recent studies (Cui et al., 2021; Li et al., 2021) have been conducted to explore interpretable discoveries from deep graph models on brain networks. However, Cui et al. (2021) focuses on explaining the message passing mechanism across the brain ROIs while ignoring the high-level network patterns within the brain networks. Li et al. (2021) tries to explain how the model generates high-level network patterns based on the graph communities. However, they only preserve the center node and discard all other nodes in the communities during the designed pooling operation.

In this work, we propose a new explainable graph representation learning framework and illustrate our method on a task predicting behavioral measures from multi-model brain connectomes in young healthy adults. We hypothesize that the intrinsic higher-level graph patterns can be preserved from the graph communities in brain networks in a hierarchical manner. Based on this assumption, we design a graph community pooling module to summarize the higher-order graph patterns. This hierarchical patterns from brain networks can be used to guide the information flow during the AI model training and increase the transparency and interpretability of the model. We demonstrate this new framework by predicting several behavioral measures using the entire brain network for each gender and investigate whether there is any significant sex difference in the results. The main contributions are summarized as follows:

- We propose a new interpretable hierarchical graph representation learning framework for brain network regression analysis.
- Comparing to state-of-the-arts methods, the regression results on Human Connectome Project (HCP) dataset demonstrate the superiority of our proposed framework.
- In order to explore the interpretability of our framework, we adopt graph saliency maps to highlight brain regions selected by the model and provide biological explanations.

## 2. Data Description

The brain network data used in this study was obtained from Zhang et al. (2020), which we summarize below. The original data was from the Human Connectome Project (HCP) 1200 Subjects Data Release (Van Essen et al., 2013). 246 region-of-interests (ROIs) from the Brainnetome atlas (Fan et al., 2016) was adopted to define the resting-state functional network and diffusion-MRI-derived structural network. Functional network was computed using CONN toolbox (Whitfield-Gabrieli and Nieto-Castanon, 2012) and structural network was processed using FSL bedpostx (Behrens et al., 2003) and probtrackx (Behrens et al., 2007). The reconstructing pipelines for these two brain networks (Ajilore et al., 2013; Zhan et al., 2015) have been described in our previous publications. In order to evaluate our framework, we selected 10 Achenbach Adult Self-Report (ASR) (Achenbach and Rescorla, 2003) measures from each subject as our prediction objectives. These 10 measures include: Anxious/Depressed Score (ANXD), Withdrawn Score (WITD), Somatic Complaints Score (SOMA), Thought Problems Score (THOT), Attention Problems Score (ATTN), Aggressive Behavior Score (AGGR), Rule Breaking Behavior Score (RULE), Intrusive Score (INTR), Internalizing Score (INTN), and Externalizing Score (EXTN). After quality control assessment of head motion and global signal changes for both scan types (diffusion MRI and resting-state fMRI) and removal of those with missing data, we included 738 young healthy subjects (mean age = 28.62±3.67, 337 males) in our study.

In sum, each subject has a 246 × 246 structural network from diffusion MRI, a 246 × 246 functional network from resting-state fMRI, and 10 ASR scores. Table 1 summarizes the ASR statistics for each gender and details of the HCP dataset can be found in footnote 1.^1^

**Table 1:** Subjects' statistics for 10 ASR scores.

**ASR score**	**Male**	**Female**	***P***
ANXD	54.58 ± 6.76	53.91 ± 6.09	1.60^−1^
WITD	54.77 ± 6.34	53.02 ± 5.32	5.38^−5^
SOMA	54.13 ± 6.05	53.97 ± 6.04	7.30^−1^
THOT	54.47 ± 5.86	53.57 ± 5.75	3.60^−2^
ATTN	55.89 ± 5.54	54.31 ± 5.68	1.55^−4^
AGGR	53.32 ± 4.83	52.47 ± 3.71	6.76^−3^
RULE	54.90 ± 6.17	53.49 ± 4.73	5.09^−4^
INTR	54.33 ± 5.95	53.27 ± 4.79	7.65^−3^
INTN	49.59 ± 11.34	48.44 ± 10.29	1.50^−1^
EXTN	50.78 ± 8.90	47.59 ± 9.04	1.85^−6^

*The two columns, corresponding to Male and Female groups, are reported as the mean ± standard deviation values. The last column is the student t-test P-value to show whether there is any significant sex difference for each ASR score*.

## 3. Methods

In this section, we first provide some preliminaries for graph learning. Then, we will explain our new framework, in which we will delve into the proposed graph pooling layer which down-scales the brain network and generates the coarse representation of brain network based on the network communities. Finally, we will briefly describe the training procedure to show that our proposed framework can be trained in an end-to-end manner.

### 3.1. Preliminaries of Graph Learning

#### 3.1.1. Graph Notation

We denote any attributed graph (i.e., brain network) with *N* nodes as *G* = (*A, X*). $A\in R^{N\times N}$ is the graph adjacency matrix saving the node connections in the graph which can be defined as:


(1) $$A_{ij}=\{\begin{array}{ll} edge\;weight & if\;node\;i\;connects\;to\;node\;j\\ 0 & otherwise. \end{array}$$

Particularly, in the functional brain networks, the edge weights measures the relationships between the BOLD signals of different brain regions (e.g., *A*_*ij*_ is the Pearson Correlation of BOLD signals between brain node *i* and *j*) (Bathelt et al., 2013; Fischer et al., 2014). By contrast, in the diffusion MRI-derived structural networks, the edge weights describe the connectivity of white matter tracts between brain regions. $X\in R^{N\times d}$ is the node feature matrix, where the dimension of the feature is *d*. We also denote $Z=\left[Z_{1:},Z_{2:},\ldots ,Z_{N:}\right]\in R^{N\times c}$ as the latent feature matrix embedded by the graph convolution layers, where *c* is the dimension of the node latent features. $Z_{i:}\in R^{1\times c}$ is the *i*-th row of matrix *Z* representing the latent feature of the *i*-th node. Given a set of labeled data $D=\left\{\left(G_{1},y_{1}\right),\left(G_{2},y_{2}\right),\left(G_{3},y_{3}\right),\ldots \right\}$ where $y_{i}\in Y$ is the regression value to the corresponding graph $G_{i}\in G$, the graph regression task is learning a mapping, f:G→Y.

#### 3.1.2. Graph Neural Network

Graph Neural Network (GNN) is an effective message-passing architecture to embed the graph nodes as well as their local structures. In general, GNN layer can be formulated as:


(2) $$\begin{array}{r} Z=F\left(A,Z;\theta \right), \end{array}$$

where θ is the trainable parameters.

*F*(·) is the forward function of GNN layer to combine and transform the messages across the graph nodes. Different expressions of *F*(·) are proposed in the previous work such as Graph Convolution Network (GCN) (Kipf and Welling, 2016) and Graph Attention Network (GAT) (Veličković et al., 2017). In this work, we adopt GCN to generate the node latent features. Following Kipf and Welling (2016), the layer of the graph neural network (i.e., Equation 2) can be instantiated as:


(3) $$\begin{array}{r} Z=\sigma \left({\tilde{D}}^{-\frac{1}{2}}Ã{\tilde{D}}^{-\frac{1}{2}}X\theta \right), \end{array}$$

where Ã = *A*+*I*, ${\tilde{D}}_{ii}=\underset{:,j}{\sum }{Ã}_{i,j}$ is the degree matrix, σ(·) is a non-linear activation function (e.g., ReLU).

### 3.2. Brain Network Representation Learning Framework

**Figure 1:**
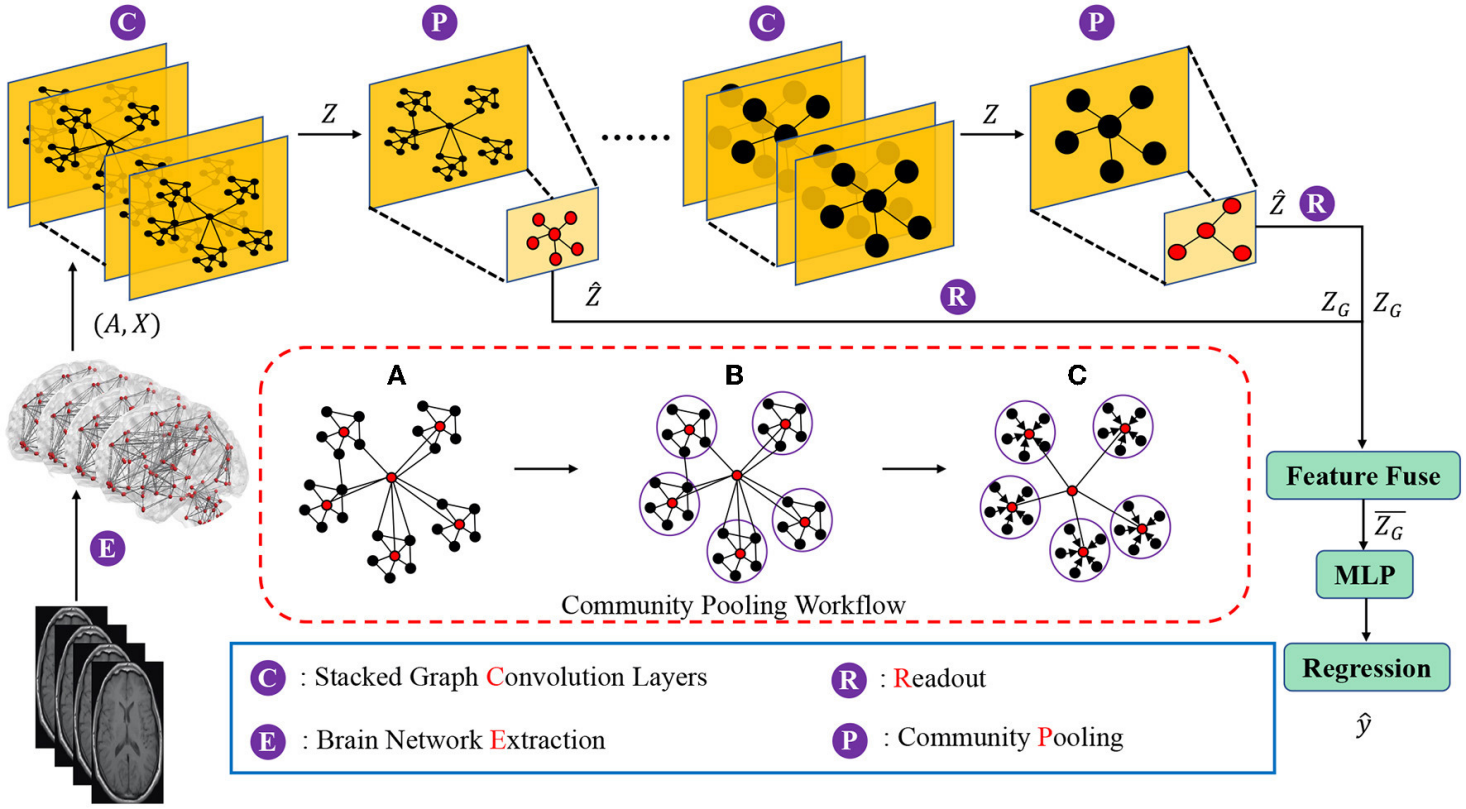
Diagram of the proposed hierarchical brain network learning framework, including stacked graph convolution layers, community pooling modules, and an Multilayer perceptron (MLP) block for the regression task. The workflow details of the proposed community pooling module are presented in the red box, including: **(A)** Compute the center node probability (P) and select the nodes with top-M P scores as center nodes. **(B)** Assign each node into the closest community. **(C)** Aggregate features of community member nodes to the corresponding center node and down scale the graph based on the captured communities.

The goal of this new brain network representation learning framework is to capture community structures of brain networks in a hierarchical manner, and to generate a representation of the whole brain network based on the preserved community information. Moreover, the proposed framework should be able to utilize derived brain network representations to achieve graph-level learning tasks (e.g., graph regression). The proposed brain network representation learning framework, as shown in Figure 1, consists of three components which are (1) nodes and local structures embedding modules, (2) community-based brain network pooling modules and (3) a task-specific prediction module. In the nodes and local structures embedding module, graph convolution layers are deployed to embed the brain network nodes and the corresponding local structures into the latent feature space. In stead of using single graph convolution layer (i.e., 1 GCN layer), we here deploy stacked graph convolution layers (i.e., stacked GCN layers, Dehmamy et al., 2019) which can promote each graph node to aggregate higher order information from a broader receptive field (i.e., to capture the information beyond one-hop neighborhoods to several-hops neighborhoods).

Given a brain network (i.e., *G* = (*A, X*)), the nodes and local structures embedding module can embed the network node features $X\in R^{N\times d}$ with its local structures $A\in R^{N\times N}$ in to the latent space as node latent features $Z\in R^{N\times c}$. The next question is that how to use these node latent features to generate the high-level graph representations? The graph convolution layers focus on the node-level representation learning and only propagate information across edges of the graph in a “flat” way (Ying et al., 2018; Tang et al., 2021). Some previous studies (Lin et al., 2013; Li et al., 2015; Vinyals et al., 2015; Zhang et al., 2018) adopted global pooling which sums, averages or concatenates all the node features as the graph-level representation and use it for graph-level tasks (e.g., graph classification, graph similarity learning). However, these methods may ignore the hierarchical structures during the global pooling process, which leads to the models ineffective in graph-level tasks. To address this issue, our proposed brain network pooling module down scales the network from *N* nodes to *M*(< *N*) nodes based on the network community which is an important graph hierarchical structures. Specifically, the proposed brain network pooling can down scale the network latent features $Z\in R^{N\times c}$ to $Ẑ\in R^{M\times c}$. Details of the proposed brain network pooling module are discussed in the next subsection.

After the network pooling, a **readout** operation is adopted to summary the whole graph representation at the current scale of the graph. Assume that we obtain the network latent feature matrix $Ẑ\in R^{M\times c}$ from the network pooling module, the readout operation generates the whole graph representation $Z_{G}\in R^{1\times c}$ by a linear layer with an activation function:


(4) $$\begin{array}{r} Z_{G}=\sigma \left(WẐ\right), \end{array}$$

where $W\in R^{1\times M}$ is the trainable parameters within the linear layer and σ(·) is an activation function (i.e., ReLu).

In the task-specific prediction module, we first fuse (e.g., concatenate, sum, average, etc.) all the graph representation *Z*_*G*_ obtained in different scales of graphs as the hierarchical graph representation for the further graph-level prediction (i.e., graph regression in this work). Then, an Multilayers Perception (MLP) is deployed to utilize the hierarchical graph representation for the graph regression task.

### 3.3. Brain Network Pooling

As mentioned before, the brain network pooling module down scales the node latent features $Z\in R^{N\times c}$ to the $Ẑ\in R^{M\times c}$ based on the network community structures. To achieve this, two basic steps are involved in the brain network pooling module including network community partition and community representation. We will discuss these two steps in sequence.

#### 3.3.1. Network Community Partition

To partition the network nodes and generate the network community, the pooling module will first identify the community center nodes and then assign other nodes to the nearest community. Inspired by the density-based partition methods (Ester et al., 1996; Heuvel van den and Sporns, 2013) that community center nodes are always densely encircled by a group of nodes with a high probability, we compute the feature distance (i.e., Euclidean distance of feature vector) as a metric to approximate the probability that measures the possibility for a node to be a center node. Specifically, a node with a smaller feature distances to all other nodes is more likely to be a community center. Based on node feature vectors, we construct the probability vector, $P\in R^{N\times 1}$ to measure the possibility that each node to be a community center node where P is formulated as:


(5) $$\begin{array}{r} P=softmax\left(1-normalize\left[\overset{N}{\underset{j=1}{\sum }}S_{i,j}\right]\right), \end{array}$$

where *S* (i.e., *S*_*i, j*_ = ||*Z*_*i*_−*Z*_*j*_||_*L*_1__) is the feature distance matrix. Finally, we select *M* nodes with Top-M P-values as *M* community center nodes.

#### 3.3.2. Community Representation

When we identify *M* community center nodes, we assign other graph nodes to the nearest the community. We denote Ω = {Ω_1_, Ω_2_, …, Ω_*M*_} as the set of all *M* communities. Then the representation of *i*-th community (i.e., Ẑ_*i*_) can be computed by:


(6) $$\begin{array}{r} {Ẑ}_{i}=Z_{c_{i}}+\underset{v_{j}\in \Omega_{i}}{\sum }Z_{v_{j}}\cdot \frac{1}{S_{i,j}}, \end{array}$$

where *Z*_*c*_*i*__ is the latent feature of the center node of *i*-th community. *v*_*j*_ are the community member nodes in the corresponding community.

### 3.4. Supervision Manner for Regression Task

As aforementioned, we fuse all graph representations *Z*_*G*_ obtained from different graph scales as the final hierarchical graph representation ${\bar{Z}}_{G}$. Then, an MLP takes ${\bar{Z}}_{G}$ as input to generate the regression prediction value ŷ. We optimize the Mean squared error (MSE) loss (i.e., ℓ_*MSE*_) to minimize the difference between the ground-truth *y* and the prediction ŷ. Meanwhile, to make the feature of community members closer to the corresponding community center node, we minimize:


(7) $$\begin{array}{r} \ell_{community}=\underset{\Omega_{i}\in \Omega }{\sum }\underset{v_{j}\in \Omega_{i}}{\sum }\ell_{MSE}\left(Z_{v_{j}},Z_{c_{i}}\right). \end{array}$$

The total loss function can be formulated as follows:


(8) $$\begin{array}{r} L_{reg}=\eta_{1}\ell_{MSE}\left(ŷ,y\right)+\eta_{2}\ell_{community}, \end{array}$$

where the η_1_ and η_2_ are the loss weights. We train the proposed brain network learning framework by minimizing this regression loss and the whole training procedure is therefore in an end-to-end manner.

## 4. Results and Discussions

### 4.1. Experiment Design and Evaluation

We will apply the proposed framework to predict ASR scores. The prediction performance will be evaluated using Mean Absolute Error (MAE). Since the community pooling module in our framework will select a group of nodes or brain regions, we can identify which brain regions (or brain network nodes) are directly linked to the prediction objects (i.e., ASR score in our study) from the last pooling module. Please be noted that this “link” doesn't mean the direct correlation since the relationship captured by our framework is non-linear by nature. We name these nodes as effecting nodes. And the last community pooling layer in our framework will generate a group of “effecting” nodes. Due to the individual difference, the effecting nodes for each subject are not exact the same. Then we count how many times each node is selected as the effecting node during the testing and normalize this number by the total number of testing subject in each group. The resulted number will be treated as the frequency of this node to be the effecting node. As a result, we can get the nodal frequency distribution for each group (male or female). Then the normalized mutual information (NMI) is used to quantify the group difference between male and female and we adopt permutation approach to evaluate the significance of the group difference.

### 4.2. Experiment Setting

For each prediction task, we randomly split the entire dataset into five disjoint sets for 5-fold cross-validations. All the prediction accuracy are calculated as the mean ± standard deviation values obtained from these 5 folders. We utilize the diffusion MRI-derived brain structural networks as the adjacency matrix input of our framework. We treat each row in the resting-state functional network as the feature for each node, so the initial nodal feature dimension is 246. We also consider using Principal Component Analysis (PCA) to reduce the nodal feature dimension. During the training stage, we optimize the parameters in the framework using the Adam optimizer (Kingma and Ba, 2015) with a batch size of 256. The initial learning rate is set to 0.001 and decayed by ${\left(1-\frac{current\text{\_}epoch}{max\text{\_}epoch}\right)}^{0.9}$. We also regularize the framework training with an *L*_2_ weight decay of 1*e*^−5^. Following the previous studies (Shchur et al., 2018; Lee et al., 2019), we adopt an early stopping criterion if the validation loss did not improve for 20 epochs in an epoch termination condition with a maximum of 500 epochs. We implement all experiments based on PyTorch (Paszke et al., 2019) and the torch-geometric graph learning library (Fey and Lenssen, 2019). All the experiments are deployed on 1 NVIDIA TITAN RTX GPUs.

### 4.3. Prediction Performance

In this section, we put all subjects (male and female) into one group and apply our method to predict ASR scores. We compare the prediction performance of our framework with 7 baseline methods to show the superiority of our framework. Two dimension reduction methods [i.e., PCA and Spectral Clustering (Ng et al., 2002) with linear regression] and five graph neural network (GNN) based models [i.e., Stacked GCN with Global-POOL, SAG-POOL (Lee et al., 2019), DIFFPOOL (Ying et al., 2018), HGP-SL (Zhang et al., 2019c) and StructPOOL (Yuan and Ji, 2020)] with different pooling layers are set as our compared baselines. The GNN based models can co-embed the brain structural networks (i.e., as adjacency matrices) and brain functional networks (i.e., as node feature matrices) into the latent space, however, two dimension reduction methods can only analyze one type of brain networks. To make a fair comparison, we only utilize brain structural networks to present the regression performance here in Table 2. Particularly, we conduct two dimension reduction methods on the brain structural networks to reduce the network dimension. Then, the linear regression is adopted on the dimension reduced networks for the regression task. Meanwhile, for the 5 GNN-based baseline models as well as ours, we initialize the node feature matrix by using all-ones vector (i.e., $\vec{1}\in R^{N\times 1}$) and only utilize the brain structural networks as the adjacency matrices. For the 5 hierarchical graph pooling models (i.e., SAG-POOL, DIFFPOOL, HGP-SL, StructPOOL and ours), we deployed 3 hierarchical graph pooling modules. Table 2 shows that our proposed framework achieves the best performance with a lowest regression Mean Absolute Error (MAE) comparing to all other methods. Meanwhile, the GNN-based methods are generally superior to the dimension reduction ones. This may result from that GNN-based methods can better extract the network local and global topological structures which are important to represent the brain networks. Moreover, the group of hierarchical graph pooling models perform better than the global pooling method, which may be explained by that our hierarchical pooling method can not only extract the graph local structures as the low-level features but also preserve these low-level features into the high level space in an hierarchical manner, while the global pooling method can only extract the graph low-level features and combine these features in a naive way (e.g., by concatenating, averaging, etc.).

**Table 2:** Regression Mean Absolute Error (MAE) with corresponding standard deviations under five-fold cross-validation on 10 ASR scores.

	**PCA+LR**	**SC+LR**	**GCN-GlobalPOOL**	**SAG-POOL**	**DIFFPOOL**	**HGP-SL**	**StructPOOL**	**Ours**
ANXD	3.66 ± 0.0083	3.52 ± 0.0004	3.01 ± 0.0013	2.26 ± 0.0071	2.01 ± 0.0021	1.78 ± 0.0062	2.11 ± 0.0012	1.49 ± 0.0033
WITD	3.07 ± 0.0005	3.19 ± 0.0083	2.81 ± 0.0055	1.87 ± 0.0052	1.91 ± 0.0008	1.69 ± 0.0049	1.94 ± 0.0036	1.18 ± 0.0011
SOMA	2.96 ± 0.0091	3.03 ± 0.0019	3.11 ± 0.0075	1.71 ± 0.0008	1.83 ± 0.0041	1.88 ± 0.0027	1.63 ± 0.0007	1.16 ± 0.0021
THOT	3.51 ± 0.0010	3.24 ± 0.0022	3.09 ± 0.0004	2.19 ± 0.0037	2.07 ± 0.0027	2.04 ± 0.0079	2.13 ± 0.0020	1.31 ± 0.0006
ATTN	3.87 ± 0.0056	3.60 ± 0.0008	2.94 ± 0.0016	2.78 ± 0.0024	2.44 ± 0.0053	2.33 ± 0.0062	2.04 ± 0.0014	1.84 ± 0.0041
AGGR	2.41 ± 0.0065	2.21 ± 0.0072	2.37 ± 0.0022	1.94 ± 0.0080	1.61 ± 0.0034	1.59 ± 0.0050	1.61 ± 0.0033	1.16 ± 0.0091
RULE	2.99 ± 0.0044	2.87 ± 0.0084	2.80 ± 0.0009	1.85 ± 0.0059	2.00 ± 0.0020	1.74 ± 0.0040	1.89 ± 0.0019	1.49 ± 0.0008
INTR	3.04 ± 0.0009	3.20 ± 0.0031	2.76 ± 0.0053	2.06 ± 0.0064	1.98 ± 0.0037	1.69 ± 0.0009	1.59 ± 0.0020	1.21 ± 0.0037
INTN	2.87 ± 0.0062	3.01 ± 0.0039	2.61 ± 0.0046	2.17 ± 0.0077	2.14 ± 0.0040	2.15 ± 0.0025	2.04 ± 0.0054	1.27 ± 0.0020
EXTN	3.70 ± 0.0017	3.54 ± 0.0055	3.45 ± 0.0071	1.98 ± 0.0034	2.22 ± 0.0005	2.07 ± 0.0037	1.98 ± 0.0018	1.58 ± 0.0012
Overall	4.62 ± 0.0038	4.37 ± 0.0018	4.02 ± 0.0045	3.62 ± 0.0029	3.39 ± 0.0088	3.05 ± 0.0011	3.24 ± 0.0013	2.93 ± 0.0084

*Overall denotes the task of jointly predicting all the 10 ASR scores. LR and SC represent linear regression and spectral clustering respectively. The values in red show the best results*.

### 4.4. Loss Weights Analysis

**Figure 2:**
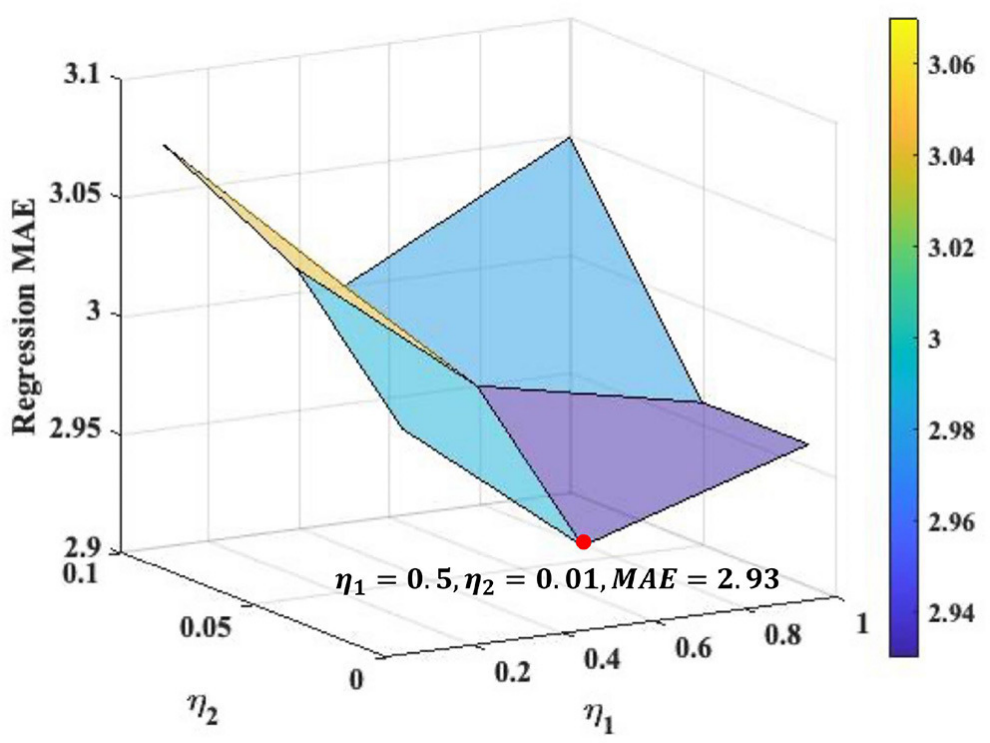
Loss weights analysis for the Overall ASR regression task. The optimal values of η_1_ and η_2_ are 0.5 and 0.01, respectively, where the MAE of overall regression achieves as 2.93.

We search the loss weights of η_1_ and η_2_ in range of [0.1, 0.5, 1] and [0.01, 0.05, 0.1], respectively, (see Figure 2) for the Overall ASR regression. The best loss weights are determined as η_1_ = 0.5 and η_2_ = 0.01. Figure 2 indicates that the performance of our framework is relatively consistent under different loss weights. We use the same loss weights setting for each single ASR prediction, although the optimal loss weights may slightly different for different prediction.

### 4.5. Impact of Community Pooling Modules on the Prediction Performance

**Figure 3:**
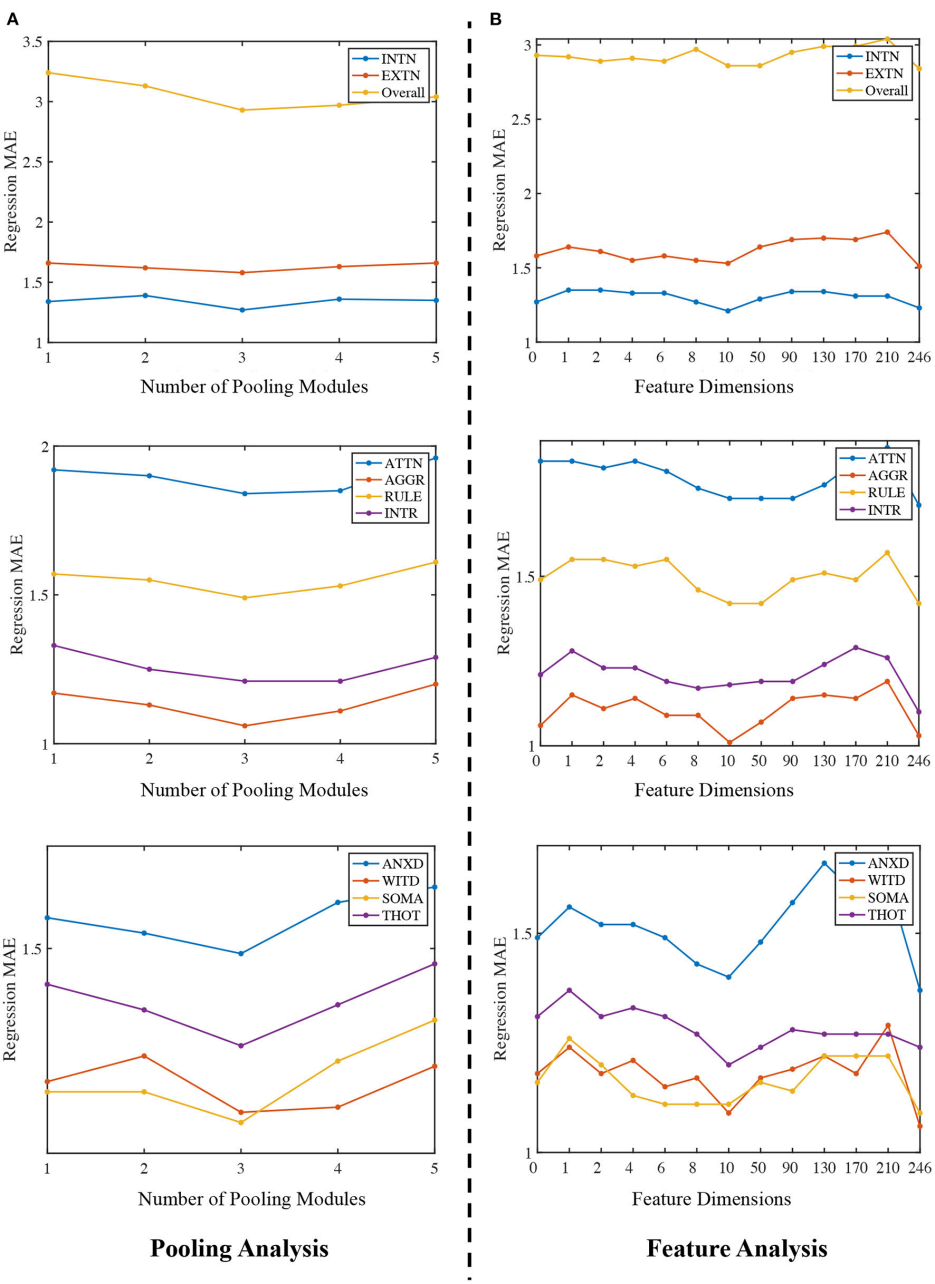
Ablation study. **(A)** Regression MAE under different number of pooling modules. The x-axis is 1 to 5, representing the number of community pooling modules and y-axis is the corresponding MAE. **(B)** Regression MAE obtained by the proposed framework when using different number of node features. The x-axis ranges from 0 to 246, representing different number of nodal features and y-axis is the corresponding MAE.

In this section, we evaluate how the number of Community Pooling modules affect the prediction performance on 10 ASR scores. We deployed different number of pooling modules (i.e., from 1 to 5) and set the pooling ratio in each pooling module as 0.5 (i.e., only 50% nodes will be preserved after each pooling module). The MAE of ASR scores obtained by the proposed framework with different number of pooling modules are shown in the Figure 3A. Figure 3A shows that the regression performance obtained by our proposed framework are consistent among different ASR scores. In general, with the increasing number of pooling modules, the MAE values first decline and then incline with the minimum MAE value is achieved when 3 pooling modules are deployed. The possible explanation is as follows: when the number of pooling modules is insufficient (e.g., 1 or 2), the high-level features related to the prediction object haven't been extracted enough; while when too many pooling modules (e.g., 4 or 5) are deployed, the extracted features may be too “coarse”, where the key discriminative information have been mosaicked.

### 4.6. Impact of Nodal Features on the Prediction Performance

Firstly, the number of the pooling modules is fixed as 3 for all experiments in this section. Then, we predict the ASR scores without using any nodal features and treat the feature dimension as zero. This is implemented by setting the node feature matrix as $\vec{1}$). After that, we use PCA algorithm to extract different number of features (from 1 to 240) and use them as the nodal features for the predictions. Lastly, we directly apply the functional network as the nodal feature matrix for the same tasks and in this situation, feature dimension is 246. Therefore, we can compare how the number of nodal features affect the prediction performance, and our results are summarized in the Figure 3B.

There are two main findings in Figure 3B. Firstly, the proposed framework can generally achieve better prediction performance by using the functional network as the node feature matrix. Secondly, we expected that using the principle components of the functional networks as the nodal features could further improve the regression or prediction performance. Among the feature dimension range from 1 to 240, the best result (i.e., the lowest MAE) is achieved at 10, in other words, using the top 10 PCs to form the feature matrix can achieve the best performance when compared with other dimension options. Moreover, although the performance obtained with 10 PCs is close to that obtained by using full functional networks (dimension = 246), using full functional network as the feature matrix (dimension = 246) generally has a better prediction performance than using PCs as the feature input, which indicate there the topological structures in the full functional networks may not be well preserved in the PCA processing. There may have some better choices for the nodal features or dimension reduction techniques, which will be considered in our future research.

### 4.7. Biological Application and Algorithm Fairness

In this section, we will demonstrate how to apply this new framework to identify sex differences. Here, sex is referred as the biological sex, as available data does not permit us to disentangle the influence of social culturally defined gender influences from biological sex effect.

We firstly apply our framework to predict each of the ASR scores for each sex. Table 3 summarizes the estimation errors (mean ± standard deviation) for each gender (column 1 and 2 for male and female respectively). Column 3 in Table 3 shows the student *t*-test *P*-values for evaluating whether there is any significant difference in the estimation errors between sexes. None of these are significant, in other words, these results demonstrates the fairness of our framework in terms of the variable “sex”.

**Table 3:** Estimation errors for predicting each ASR score for each gender.

**ASR score**	**Male**	**Female**	***P***
ANXD	1.74 ± 0.03	1.73 ± 0.02	0.66
WITD	1.24 ± 0.02	1.24 ± 0.03	0.82
SOMA	1.25 ± 0.02	1.27 ± 0.06	0.44
THOT	1.45 ± 0.05	1.40 ± 0.04	0.10
ATTN	1.96 ± 0.06	1.95 ± 0.03	0.78
AGGR	1.26 ± 0.04	1.24 ± 0.03	0.31
RULE	1.62 ± 0.07	1.55 ± 0.08	0.16
INTR	1.37 ± 0.05	1.35 ± 0.05	0.47
INTN	1.37 ± 0.08	1.32 ± 0.08	0.38
EXTN	1.64 ± 0.09	1.71 ± 0.18	0.43

*The results are reported in the format of mean ± standard deviation. The last column is the Student t-test P-value to show whether there is any significant difference in the estimation errors between male and female. These results indicate that our new framework is fair for the variable “sex”*.

**Figure 4:**
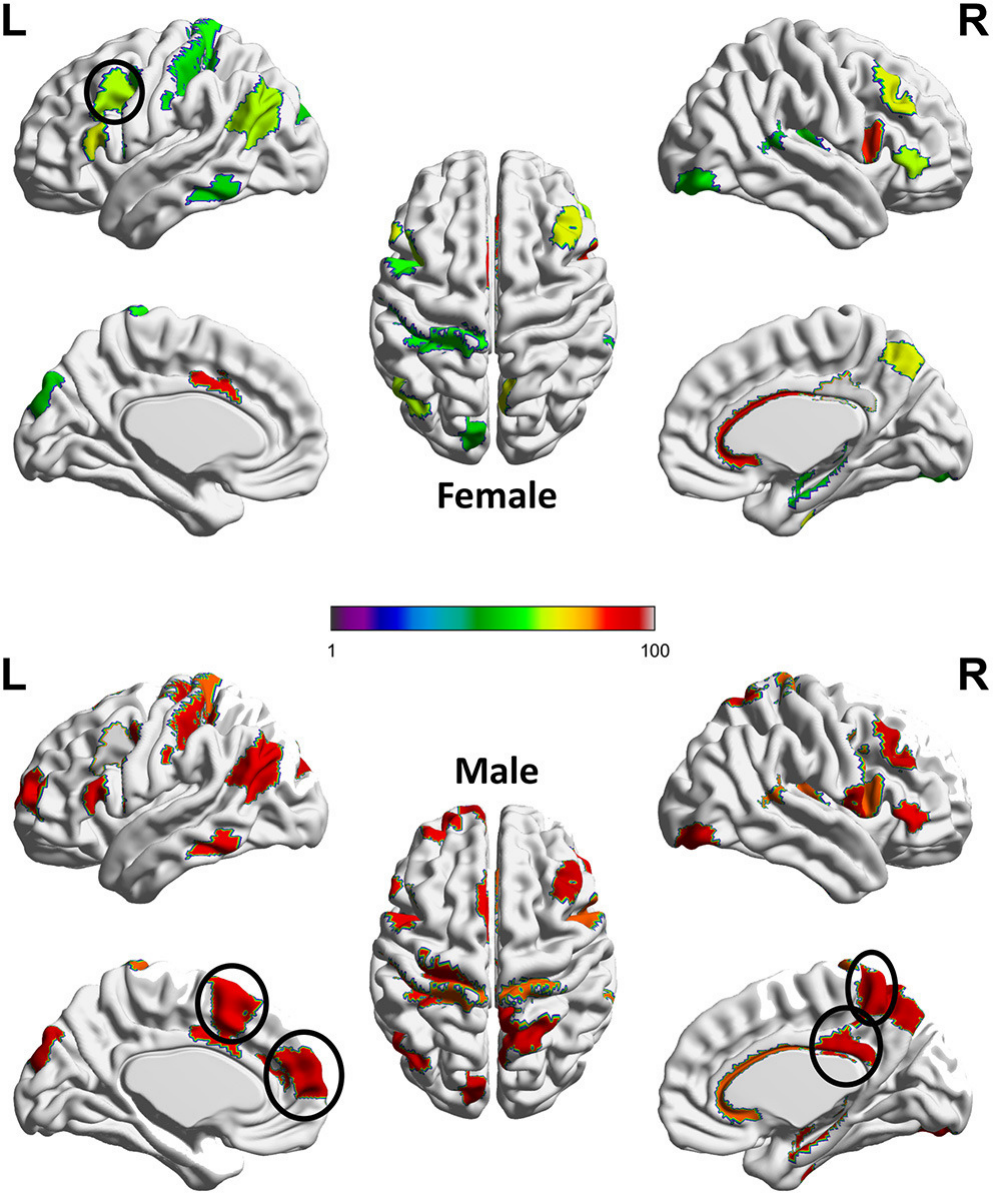
Sex difference identified for ATTN. The color indicates the region involved in the ATTN process and the hotter color indicate the stronger involvement and the cooler color indicate the inverse. Permutation tests have been adopted to confirm the significance of this sex difference (*p* < 0.01).The main sex differences are in several regions, which are highlighted using a black circle. These regions include Left Paracentral lobule, Right Posterior cingulate and Left dorso-medial prefrontal cortex, Right Precuneus, and Left Premotor.

Next, we adopt the permutation approach to evaluate whether there are significant sex differences in the “effecting” node distributions for each ASR score (Please refer to Section 4.1 for technique details). We randomly shuffle the subjects between male and female groups and conduct 100 permutations. All permutation tests are conducted using the computation resource in the Pittsburgh Supercomputing Center (PSC) (Towns et al., 2014; Nystrom et al., 2015). Our permutation results show that there are significant sex differences (*p* < 0.01) in the effecting node distributions for 7 ASR variables except ANXD, SOMA and INTN, which is consistent with the conclusions from Table 1. Here we choose ATTN as an example to show the sex differences in the effecting nodal distribution. Attention problem score (ATTN) (Achenbach and Rescorla, 2003) indicates the tendency to be easily distracted and unable to concentrate more than momentarily. Figure 4 shows the effecting node distributions for male and female, and the hot color indicates the stronger involvements of that ROI in this psychiatric process (or ATTN) and the cool color indicate the opposite. Our results show there are multiple brain regions (including Left Paracentral lobule, Right Posterior cingulate and Left dorsomedial prefrontal cortex, Right Precuneus, and Left Premotor, highlighted using black circle in Figure 4) showing significantly different involvements in this psychiatric process between sexes.

Previous studies reported that paracentral lobule is activated in covert shifts of attention (Grosbras et al., 2005) and auditory attention shifting (Huang et al., 2012). Moreover, Dickstein et al. (2006) reported that right paracentral lobule had a greater probability of activation in patients with Attention-deficit/hyperactivity disorder (ADHD) than in controls while our results show that part of sex differences for healthy controls is in the left paracentral lobule, which deserves further investigations in the future. The posterior cingulate cortex (PCC) is a central node of the default mode network (DMN) and many evidence suggests that the PCC plays a direct role in attentionally demanding tasks (Gusnard and Raichle, 2001; Vogt and Laureys, 2005; Hampson et al., 2006; Hahn et al., 2007; Leech et al., 2011; Leech and Sharp, 2014). The dorsomedial prefrontal cortex (dmPFC) receives afferent input from sensory and parietal regions of the cortex, which presumably enable the dmPFC to respond to situations that require immediate attention and respond with appropriate actions (Narayanan and Laubach, 2006; Venkatraman et al., 2009; Park et al., 2016). Additionally, Precuneus has been reported to highly involve in attention shift (Cavanna and Trimble, 2006) while Premotor is involved in Reorienting attention (Rizzolatti et al., 1987) and attention-deficit/hyperactivity disorder (Mostofsky et al., 2002). All these clearly indicate that our new AI framework can discover potential biologically-meaningful results for regression studies.

## 5. Conclusion

In this study, we proposed a novel interpretable graph learning framework for brain network regression analysis. We demonstrated that our new framework has better prediction performances than state-of-the-arts graph learning methods in predicting young health subjects' psychiatric scores. Additionally, we chose one of the psychiatric scores to demonstrate how this new framework can be used to study sex differences. Future work will focus on how to modify our framework for the signed graph data.

## Data Availability Statement

Publicly available datasets were analyzed in this study. This data can be found here: https://www.humanconnectome.org/study/hcp-young-adult/document/1200-subjects-data-release.

## Author Contributions

HT took charge of conception and design, method implementation, statistical analysis, and interpretation, as well as manuscript writing and revising. LZ took charge of project design, data preprocessing, analysis and interpretation, manuscript writing/revising. LG, XF, BQ, OA, YW, PT, HH, and AL took charge of experiment design, results discussion, and manuscript proofreading. All authors contributed to the article and approved the submitted version.

## Funding

This study was partially supported by the National Institutes of Health (R01AG071243, R01MH125928, and U01AG068057) and National Science Foundation (IIS 2045848 and IIS 1837956).

## Conflict of Interest

The authors declare that the research was conducted in the absence of any commercial or financial relationships that could be construed as a potential conflict of interest.

## Publisher's Note

All claims expressed in this article are solely those of the authors and do not necessarily represent those of their affiliated organizations, or those of the publisher, the editors and the reviewers. Any product that may be evaluated in this article, or claim that may be made by its manufacturer, is not guaranteed or endorsed by the publisher.

## Footnotes

^1^ https://wiki.humanconnectome.org
